# Cell-to-Cell Propagation of the Bacterial Toxin CNF1 via Extracellular Vesicles: Potential Impact on the Therapeutic Use of the Toxin

**DOI:** 10.3390/toxins7114610

**Published:** 2015-11-05

**Authors:** Alessia Fabbri, Sara Cori, Cristiana Zanetti, Marco Guidotti, Massimo Sargiacomo, Stefano Loizzo, Carla Fiorentini

**Affiliations:** 1 Department of Therapeutic Research and Medicines Evaluation, Istituto Superiore di Sanità, Rome 00161, Italy; E-Mails: alessia.fabbri@iss.it (A.F.); sara_cori@hotmail.it (S.C.); stefano.loizzo@iss.it (S.L.); 2 Department of Hematology, Oncology and Molecular Medicine, Istituto Superiore di Sanità, Rome 00161, Italy; E-Mails: cristiana.zanetti@iss.it (C.Z.); massimo.sargiacomo@iss.it (M.S.); 3 Department of Veterinary Public Health and Food Safety, Istituto Superiore di Sanità, Rome 00161, Italy; E-Mail: marco.guidotti@iss.it

**Keywords:** extracellular vesicles, toxin transfer, intercellular communication, bacterial toxin, CNF1

## Abstract

Eukaryotic cells secrete extracellular vesicles (EVs), either constitutively or in a regulated manner, which represent an important mode of intercellular communication. EVs serve as vehicles for transfer between cells of membrane and cytosolic proteins, lipids and RNA. Furthermore, certain bacterial protein toxins, or possibly their derived messages, can be transferred cell to cell via EVs. We have herein demonstrated that eukaryotic EVs represent an additional route of cell-to-cell propagation for the *Escherichia coli* protein toxin cytotoxic necrotizing factor 1 (CNF1). Our results prove that EVs from CNF1 pre-infected epithelial cells can induce cytoskeleton changes, Rac1 and NF-κB activation comparable to that triggered by CNF1. The observation that the toxin is detectable inside EVs derived from CNF1-intoxicated cells strongly supports the hypothesis that extracellular vesicles can offer to the toxin a novel route to travel from cell to cell. Since anthrax and tetanus toxins have also been reported to engage in the same process, we can hypothesize that EVs represent a common mechanism exploited by bacterial toxins to enhance their pathogenicity.

## 1. Introduction

An essential hallmark of multicellular organisms is the ability of cells to communicate with each other, an intercellular cross-talk that has been so far principally ascribed to a direct cell-to-cell contact or to the action of secreted molecules. In the last two decades, a third cell-to-cell communication mechanism of eukaryotic cells, involving intercellular transfer of extracellular vesicles (EVs), has emerged [1]. EVs are released into the extracellular environment essentially by three different processes: (i) the production of exosomes that stem from the exocytic fusion of multivesicular bodies (MVBs); (ii) the formation of vesicles that sprout from the plasma membrane, resulting in ectosomes or plasma membrane-derived vesicles; and (iii) the emergence of apoptotic blebs from dying cells [2]. Exosomes are characterized by a cup-shaped morphology with a diameter of 20–100 nm, whereas ectosomes display a heterogeneous morphology and are larger in diameter (100–1000 nm) [3].

The communication route via EVs is extensively employed in both eukaryotic and prokaryotic worlds. Bacteria, fungi and parasites have been reported to use, as a pathogenic mechanism, the release of bioactive molecules via vesicles. In this context, it has been demonstrated that the entry of “outer membrane vesicles” derived from *Escherichia coli* into the bloodstream induces systemic inflammation mimicking sepsis [4] and that *Staphylococcus aureus*-derived “cytoplasmic membrane vesicles” are related to the pathogenesis of atopic dermatitis-like inflammation and neutrophilic pulmonary inflammation [5,6]. Regarding bacterial toxin internalization by eukaryotic cells, it has been reported that anthrax toxin and tetanus toxin, both protein toxins that have to be internalized in order to act on a cytosolic target (the so-called A-B toxins, A for the catalytic domain and B for the binding domain) [7], once inside an eukaryotic cell, can be delivered to the extracellular medium as EVs [8,9].

In the last few years, the possibility of re-engineering naturally-derived EVs for targeted gene therapy has emerged as an alternative therapeutic approach. EVs represent an ideal delivery vector for different reasons: (i) they are completely natural, non-synthetic and non-viral; (ii) their size and plasticity makes it possible to cross biological membranes; and (iii) their lipid structure, besides enabling the delivery to their target, preserves the degradation of the cargo [10].

In this context, we investigated whether the *E. coli* A-B protein toxin named cytotoxic necrotizing factor 1 (CNF1) could be delivered by eukaryotic cells to the extracellular medium via EVs. CNF1 constitutively activates the ubiquitously-expressed regulatory proteins that belong to the Rho GTPases family (encompassing the three subfamilies Rho, Rac and Cdc42). Such activation occurs through deamidation of a critical glutamine residue that locks them in their activated, GTP-bound state [11,12]. In recent years, CNF1 has been proposed as a novel potential therapeutic tool for a number of central nervous system (CNS) diseases [13]. In fact, a direct brain injection of CNF1 is able to reverse cognitive impairment and neuroinflammation in Rett syndrome and Alzheimer’s disease mouse models [14,15]. The systemic administration of EVs (able to cross the BBB) containing CNF1 or the signal fostered by the toxin could represent a valid alternative for a less invasive administration route.

To achieve this target, the first step has been to verify if EVs derived from eukaryotic cells challenged with CNF1 could induce, in cultured cells, effects comparable to those caused by CNF1 itself. The results obtained confirmed this hypothesis, clearly demonstrating that EV-CNF1 activates Rac1, reorganizes the actin cytoskeleton and induces NF-κB nuclear translocation.

## 2. Results

### 2.1. EV-CNF1 Induces Cytoskeletal Changes and Rac1 Activation

**Figure 1:**
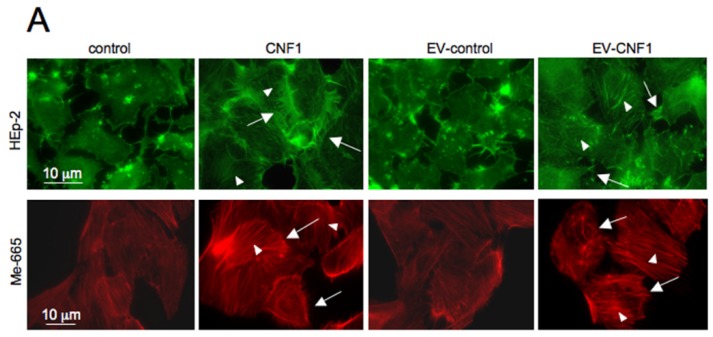

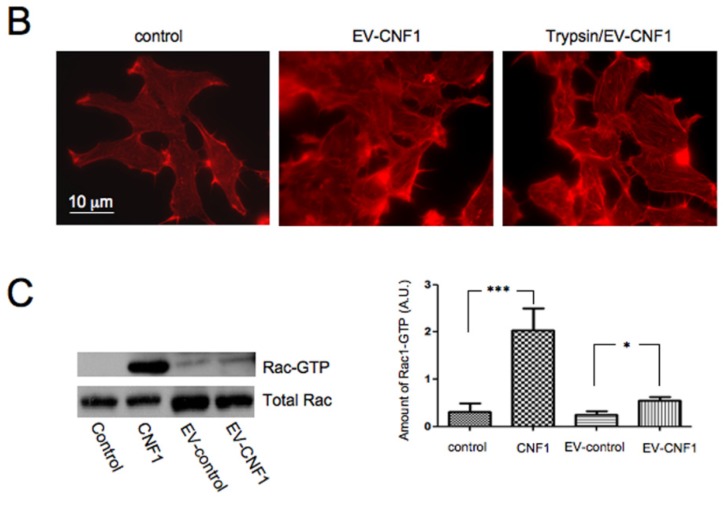
Actin cytoskeleton modification and Rac1 activation induced by extracellular vesicles (EVs) in cells. (**A**) Fluorescence micrographs of HEp-2 and Me-665 cells stained with fluorescein- or Tetramethylrhodamine (TRITC)-phalloidin to detect the actin cytoskeleton organization. Cells exposed to EVs derived from CNF1-treated cells for 24 h (EV-CNF1) show a rearrangement of the actin cytoskeleton in stress fibers (arrowheads) and ruffles/spikes (arrows) similar to that obtained in CNF1-treated cells. (**B**) Fluorescence micrographs of HEp-2 cells stained with TRITC-phalloidin to detect the actin cytoskeleton organization. Cells exposed to EV-CNF1 derived from trypsin-treated cells show a rearrangement of the actin cytoskeleton in stress fibers and ruffles/spikes similar to that obtained in cells exposed to EV-CNF1. (**C**) Western blot analysis of the pull-down assay of HEp-2 cells showing the increase in Rac1-GTP following treatment with CNF1, as well as with EV-CNF1. The blot in the left panel shows one representative experiment, whereas the graph in the right panel reports the mean ± SEM from three different experiments (*n* = 3 experiments, with each experiment performed in duplicate). * *p* < 0.05; *** *p* < 0.001.

In order to verify if exosomes and/or ectosomes could carry and propagate CNF1 (or its specific signals) from cell to cell, we first verified, by fluorescence microscopy, the actin cytoskeleton organization in cells challenged either with CNF1, or with EVs from control cells (EV-control), or derived from cells pre-exposed to CNF1 for 2 h (EV-CNF1). As shown in Figure 1A, incubation of cells with EV-CNF1 drove actin cytoskeleton changes that morphologically resemble those induced by CNF1 itself, mainly consisting of the formation of stress fibers (arrowheads) and ruffling/spikes (arrows). This result supports the hypothesis that EV-CNF1 can carry the CNF1 activity. Furthermore, we also tested the EV-CNF1 in an additional cell type, the human metastatic cell line 665 (Me-665) that has been shown to be suitable for studies on toxins and exosomes [16]. The cytoskeletal changes induced by EV-CNF1 were comparable to that provoked by the toxin (Figure 1A), indicating that EV-CNF1 could represent a general route of toxin propagation not restricted to a specific cell type. When an activity assay was performed, by comparing titration of CNF1 with EV-CNF1 effects on actin in HEp-2 cells, we found that the response obtained with EV-CNF1 corresponded to that obtained with 0.7 × 10^−12^ M CNF1. Furthermore, to eliminate the possibility that a small amount of CNF1, eventually present in the medium used to collect the EVs, could be responsible for the observed activity, we briefly treated cells with trypsin before overnight incubation. As shown in Figure 1B, treatment of cells producing EVs with trypsin did not inhibit the capability of EV-CNF1 to rearrange the actin cytoskeleton, proving that CNF1 is inside the EVs.

To further support that EV-CNF1 can carry the CNF1 activity, we performed pull-down experiments of Rac1-GTP to verify whether EV-CNF1 could also activate Rac1, the Rho GTPase whose activation is predominant in CNF1-treated HEp-2 cells [17] and in mouse brains, as well [18,19,20]. When compared with their respective controls, the intensities of the Rac1-GTP bands were increased in cells treated with the toxin, as well as in cells exposed to EV-CNF1 (Figure 1C).

### 2.2. EV-CNF1 Stimulates NF-κB Nuclear Translocation

**Figure 2:**
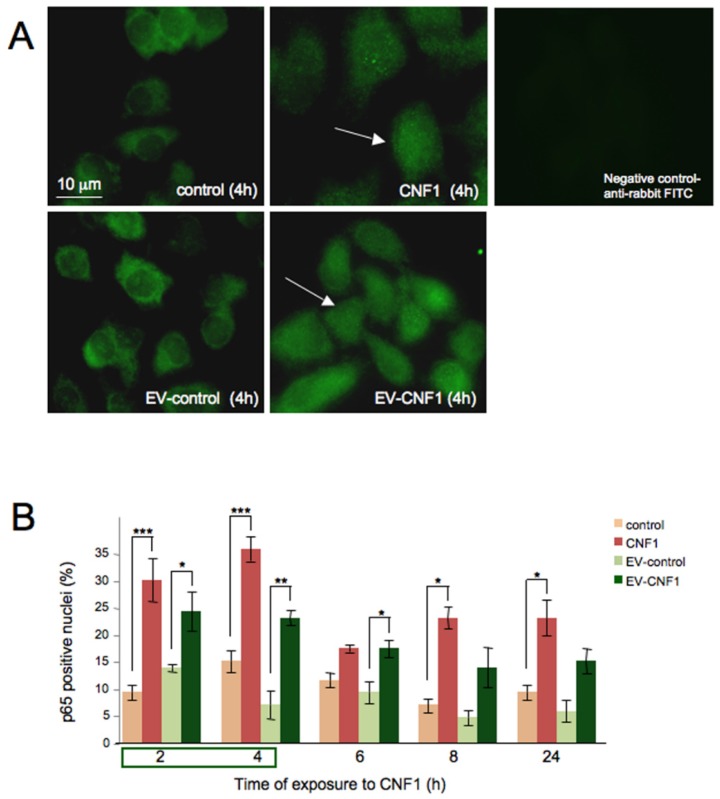
NF-κB translocation in EV-CNF1-treated cells. (**A**) Fluorescence micrographs of HEp-2 cells untreated or treated for 4 h with CNF1 or with EV-CNF1, stained with an anti-p65 antibody. The illustrations are used to evidence the positively-stained nuclei (arrows) after CNF1 or EV-CNF1 exposure. The negative staining for rabbit antibody is shown. (**B**) The graph reports the mean ± SEM from three different experiments (*n* = 3 experiments, with each experiment performed in duplicate), showing the time-dependent nuclear translocation of p65 NF-κB after the different experimental conditions. * *p* < 0.05; ** *p* < 0.01; *** *p* < 0.005.

The second step was to verify if EV-CNF1 could induce another typical activity stimulated by CNF1 in HEp-2 cells: that is, the nuclear translocation of the transcription factor NF-κB [21,22]. Such translocation occurs maximally between 4 and 10 h from the treatment with CNF1, with about 50% of positive nuclei for the p65 protein [21]. We herein investigated whether EV-CNF1 could trigger a response similar to that of CNF1. Immunofluorescence micrographs (Figure 2A) revealed that in both cells treated with the toxin or with EVs taken from cells previously intoxicated with CNF1 for 2 h, the p65 NF-κB subunit was translocated into the nucleus. Moreover, when a quantitative analysis was performed (Figure 2B), we found a nearly comparable percentage of cells with p65-positive nuclei between CNF1 and EV-CNF1. In particular, the percentage of cells with p65-positive nuclei was around 20% in EV-CNF1-treated cells, whereas cells exposed to CNF1 reached 26%–31% of positivity. The maximum nuclear translocation of p65 NF-κB was obtained between 2 and 4 h after treatment with CNF1 or EV-CNF1. Starting from 6 h of treatment, a general decrease in p65-positive nuclei was observed, which was consistent with the drop in Rho GTPase activation previously reported for this cell line [22]. The increment at 8 and 24 h of exposure with respect to 6 h was only apparent, since such an augmentation was not significant (*p* = 0.3355 by ANOVA). These results indicate that EVs are able to propagate the toxin activity, with an effect similar to that of CNF1.

### 2.3. CNF1 Is Detectable in EVs Derived from CNF1-Intoxicated Cells

**Figure 3:**
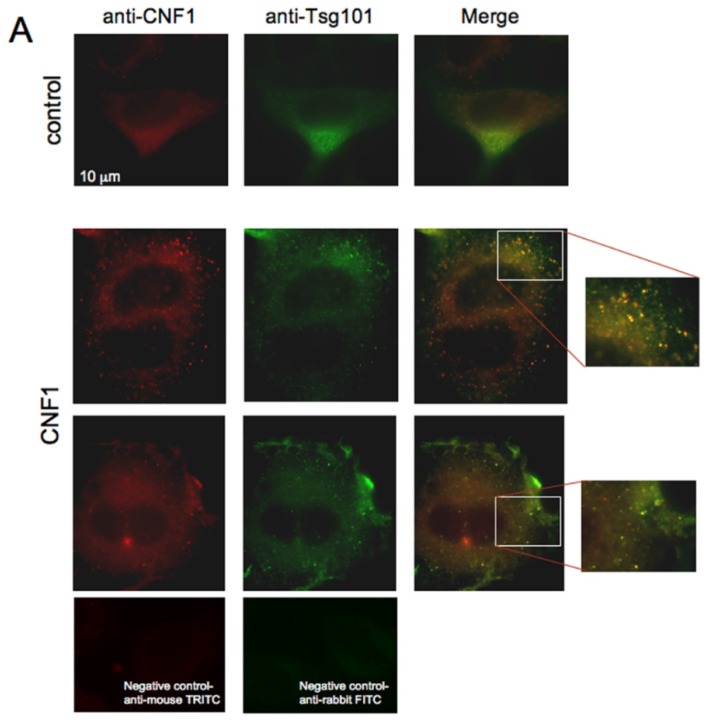

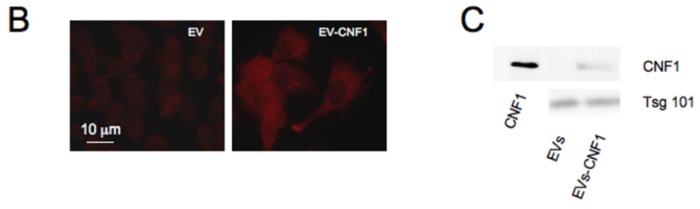
CNF1 colocalizes with Tsg101-positive vesicles and is carried by CNF1-EVs. (**A**) Fluorescence micrographs of HEp-2 cells double stained with anti-Tsg101, a marker of exosomes, and anti-CNF1. In control cells, Tsg101 is distributed inside the cytoplasm in a polarized way close to the nucleus, whereas CNF1 staining is negative. In CNF1-treated cells, a clear staining with the anti-CNF1 is observable inside the cytoplasm, as well as at the periphery of the cells. Interestingly, Tsg101 was co-localized with CNF1 in cytoplasmic punctuated structures, as well as in vesicle-like structures detected at the cell periphery (insets). Negative staining for mouse and rabbit antibodies is shown. (**B**) Fluorescence micrographs of HEp-2 cells incubated with EV and EV-CNF1 for 24 h and stained with the CNF1 antibody. In cells incubated with EV-CNF1, a positive staining is clearly evident. (**C**) Western blot analysis of EV and EV-CNF1 showing the presence of CNF1 inside EV-CNF1. Purified CNF1 was loaded as a positive marker.

The third step was to verify whether CNF1 could be found in EVs. To address this question, HEp-2 cells were treated with CNF1 for 24 h, and the presence of the toxin was verified by fluorescence microscopy, by double staining cells with anti-Tumor Susceptibility Gene (Tsg) 101, a marker for exosomes, and anti-CNF1. As shown in Figure 3, in control cells, Tsg101 was distributed in the cytoplasm in a polarized way close to the nucleus, whereas CNF1 staining was negative. In CNF1-challenged cells, the anti-CNF1 staining clearly showed the presence of the toxin inside the cytoplasm, as well as at the cell periphery. Interestingly, Tsg101 co-localized with CNF1 both in cytoplasmic punctuated structures and in the vesicle-like structures detected at the cell periphery (see the insets in Figure 3). Thus, we wondered whether CNF1 could be found in EVs derived from cells intoxicated with CNF1. To address this question, HEp-2 cells were treated with EVs or EV-CNF1 for 24 h and then stained with an anti-CNF1 antibody. As shown in Figure 3B, cells treated with EV-CNF1 were clearly positive for the anti-CNF1 staining, whereas EV-treated cells were negative. We thus verified, by Western blot analysis, whether CNF1 could be found in EVs derived from CNF1-intoxicated cells. As shown in Figure 3C, whereas EVs derived from control cells were negative for CNF1, EV-CNF1 were clearly positive for the toxin. Furthermore, we have quantified the amount of CNF1 protein alone and CNF1 protein contained in EVs that is delivered to the untreated cells. A densitometric quantification of CNF1 bands in Figure 3C revealed the presence of about 1.17 × 10^−2^ ng of CNF1 per μg of EVs. These results demonstrate that CNF1 can be found in EV derived from CNF1-intoxicated cells and strongly support the hypothesis that CNF1 can travel from cell to cell also via extracellular vesicles.

## 3. Discussion

This work shows that EVs from CNF1-treated cells are able to propagate the activity of CNF1 and that they contain the toxin itself. This novel finding demonstrates that CNF1 has a previously-uncharacterized means of propagation from cell to cell. 

EVs have already been indicated as virulence factor carriers in diseases associated with protozoa [23] and viruses [24]. Extensive studies have been also performed on outer membrane vesicles produced by bacteria that contain biologically-active proteins and perform diverse biological processes [25]. More recently, it has been reported that eukaryotic cells, once intoxicated with tetanus or anthrax toxins, can subsequently spread the toxins from cell to cell by means of EVs as vehicles. Tetanus toxin has been used as a marker of endosomes in a paper showing that differentiated neurons secrete exosomes that originate from endosomes, thus indirectly highlighting that tetanus toxin could be conveyed by exosomes [9]. Regarding anthrax toxin, the study by Abrami and coworkers [8] reveals that the uncommon intracellular route of anthrax toxin leads to the generation of a toxin pool that ensures prolonged toxicity, as well as its possible transmission to distant cells via exosomes. A mention is also due for the Staphylococcal α toxin, whose ability to permeate the plasma membrane can damage target cells. In fact, in injured cells, the toxin pores are taken up by endocytosis and sequestered in intraluminal vesicles, and finally, the lack of degradation in lysosomes leads to their discharge to the extracellular space in exosomes [26]. In the present paper, we report for the first time that CNF1 can be found inside EVs and transmitted through them. In fact, our results show that EVs derived from CNF1-treated cells can stimulate the same actin rearrangement induced by CNF1, as well as can promote Rac1 activation and NF-kB nuclear translocation. The EV-CNF1 appear to contain the toxin that, in such a way, can enhance its spreading among cells. However, we cannot rule out that, in addition to the toxin itself, also a CNF1-dependent cellular signaling molecule trapped inside the vesicles can contribute to the toxin propagation effect. These results suggest the existence of a new general strategy employed by bacterial toxins to efficiently spread from cell to cell by EVs, perhaps to increase bacterial infectivity, as well as to escape from the host immune response.

This way of toxin spreading may suggest alternative possibilities for CNF1 administration *in vivo* and may represent an important step forward from a pharmacological point of view. In fact, CNF1 has been proven to ameliorate cognitive performance if injected in the brain of animal models for Rett syndrome [14] and Alzheimer’s disease [15]. The use of EV-CNF1 for systemic administration of the toxin in the treatment of CNS diseases could overcome the direct brain administration so far employed in experimental animals.

## 4. Materials and Methods

### 4.1. CNF1 Purification

CNF1 was purified from the 392 ISS strain (a generous gift from V. Falbo, Rome, Italy), as described [27].

### 4.2. Cell Cultures and Treatments

Human epithelial HEp-2 cells (ATCC^®^ CCL23™) and melanoma Me-665 cells [28] were grown in Dulbecco’s Modified Eagle’s Medium supplemented with 10% fetal bovine serum (Flow Laboratories, Rockville, MD, USA), 5 mM l-glutamine, 100 U/mL penicillin and 100 μg/mL streptomycin. Experiments were performed with cells in exponential growth. For EVs production, HEp-2 and Me-665 cells were seeded at a density of 2 × 10^4^ cells/cm^2^, and 24 h after the seeding, cells were exposed to 10^−10^ M CNF1 for 2 h. For experiments with EVs, cells seeded as above were treated with 10 μg of EVs for 24 h.

For the activity assay, CNF1 was titrated on HEp-2 cells with serial dilution starting from 10^−9^ M to 10^−14^ M CNF1.

### 4.3. Collection of Extracellular Vesicles

Cells incubated with CNF1 for 2h at 37 °C were extensively washed with 1 × PBS and incubated for 24 h with CNF1-free medium with 10% fetal bovine serum (previously deprived of bovine microvesicles by ultracentrifugation for 6 h at 100,000× *g*). EVs were collected from the culture supernatants of CNF1-treated or untreated HEp-2 and Me-665 cells after 24 h. EV collection was performed by differential centrifugations, as previously described [29,30], with minor modifications. Briefly, culture supernatants were centrifuged at 2000× *g* for 20 min and then at 10,000× *g* for an additional 20 min, followed by filtration through a 0.22-um filter. EVs were then pelleted by ultracentrifugation (Beckman Ti SW41 rotor, Palo Alto, CA, USA) at 100,000× *g* for 4 h and washed once in PBS. The obtained EVs were resuspended into PBS and stored at +4 °C until use. For experiments aimed at eliminating unbound CNF1, after 2 h of incubation with CNF1, cells were extensively washed as described and incubated with trypsin (0.25% *v*/*v*) for 3 min. After washing, cells were incubated for 24 h and processed for EVs collection, as described above.

### 4.4. Amount Determination of the Collected EVs

EV concentration was determined by analyzing protein concentration using the Bio-Rad protein quantification assay kit (Bio-Rad Laboratories, Inc., Richmond, CA, USA) with bovine serum albumin as a standard.

### 4.5. Western Blot Analyses and Pull-Down Assay

Thirty micrograms of EVs, obtained as described above, were resuspended in 50 mM Tris/HCl, pH 6.8, 2% SDS, 10% glycerol, 100 mM dithiothreitol and processed for Western blotting, as previously described [22]. As primary antibodies, monoclonal anti-CNF1 (Abcam, Cambridgeshire, UK) and monoclonal Tsg101 (GeneTex, San Antonio, TX, USA) were used.

Pull-down assays were performed as described previously [31]. Briefly, cells lysed in the appropriate buffer were incubated with 80 μg of GST-PAK CD fusion protein (prepared as described previously in [31]). After washing in the appropriate buffer, the bound proteins were eluted in sample buffer and subjected to SDS-PAGE and immunoblotting with the mouse monoclonal anti-Rac1 (1:3500; BD Biosciences Transduction Laboratories, Lexington, KY, USA). Whole-cell lysates were analyzed in parallel. Autoradiographs were scanned, and signal quantification was performed using the NIH ImageJ software (Bethesda, MD, USA) and normalized as a function of the total proteins loaded in the assay.

### 4.6. Immunofluorescence Microscopy

To visualize F-actin organization, cells were first fixed in 3.7% paraformaldehyde in PBS and then permeabilized with 0.5% Triton X-100. After washing in PBS, coverslips were stained with FITC- or TRITC-phalloidin (Sigma, St. Louis, MO, USA) for 30 min at room temperature. Following extensive washing in PBS, cells were mounted on glass coverslips and analyzed as stated below. p65 immunostaining was obtained by fixing cells in acetone:methanol, 1:1 (*v*/*v*) for 10 min at room temperature and air dried. Cells were re-hydrated by preincubation with PBS containing 10% of AB human serum for 1 h, before being incubated with the p65 antibody (Santa Cruz, Dallas, TX, USA) for 1 h at room temperature. Following extensive washing, cells were incubated for 1 h at room temperature with FITC anti-rabbit antibody, washed, mounted on glass coverslips and analyzed as described below. For CNF1/Tsg101 staining, cells, fixed and permeabilized as described for F-actin detection, were incubated with the CNF1 antibody (Abcam) and Tsg101 antibody (Pierce) for 1 h at room temperature. Following three washes in PBS, cells were incubated for 30 min at room temperature with TRITC-labelled anti-mouse antibody followed by 3 washes in PBS and an additional 30-min incubation with an FITC-labelled anti-rabbit antibody. For CNF1 staining, cells were: (1) incubated with the CNF1 antibody; (2) rinsed in PBS; and (3) incubated with TRITC-labelled anti-mouse antibody. For negative staining, cells were incubated with total rabbit serum or anti-mouse IgG2a (Sigma), followed by the appropriate secondary antibody. Following extensive washing, coverslips were mounted and analyzed with a fluorescence microscope (Olympus BX51, Tokyo, Japan). Fluorescence analyses were performed by a charge-coupled device (CCD) camera (Carl Zeiss, Oberkochen, Germany).

### 4.7. Statistical Analysis

Data were presented as the mean ± SEM. For statistical analysis between two groups, an independent sample *t*-test was used; for more than two groups, a one-way ANOVA test was used. Means were considered as significant when the *p*-value was <0.05.

## 5. Conclusions

Taken altogether, our results reveal the existence of a novel way of propagation for CNF1 in eukaryotic cells. Since the same mechanism has been already reported for anthrax and tetanus toxins, we can hypothesize that EVs released from eukaryotic cells represent a common mechanism exploited by bacterial toxins to reinforce their pathogenic activity on the host.
